# Mitochondrial and Nuclear Genes of Mitochondrial Components in Cancer

**DOI:** 10.2174/138920209788488517

**Published:** 2009-06

**Authors:** E Kirches

**Affiliations:** Department of Neuropathology, Otto-von-Guericke University, Magdeburg, Germany

## Abstract

Although the observation of aerobic glycolysis of tumor cells by Otto v. Warburg had demonstrated abnormalities of mitochondrial energy metabolism in cancer decades ago, there was no clear evidence for a functional role of mutant mitochondrial proteins in cancer development until the early years of the 21^st^ century. In the year 2000, a major breakthrough was achieved by the observation, that several genes coding for subunits of the respiratory chain (ETC) complex II, *succinate dehydrogenase *(SDH) are tumor suppressor genes in heritable paragangliomas, fulfilling Knudson’s classical two-hit hypothesis. A functional inactivation of both alleles by germline mutations and chromosomal losses in the tumor tissue was found in the patients. Later, *SDH *mutations were also identified in sporadic paragangliomas and pheochromocytomas. Genes of the mitochondrial ATP-synthase and of mitochondrial iron homeostasis have been implicated in cancer development at the level of cell culture and mouse experiments. In contrast to the well established role of some nuclear *SDH *genes, a functional impact of the mitochondrial genome itself (mtDNA) in cancer development remains unclear. Nevertheless, the extremely high frequency of mtDNA mutations in solid tumors raises the question, whether this small circular genome might be applicable to early cancer detection. This is a meaningful approach, especially in cancers, which tend to spread tumor cells early into bodily fluids or faeces, which can be screened by non-invasive methods.

## 1. INTRODUCTION: ENERGY METABOLISM OF CANCER CELLS

### 1.1. Otto Warburg’s Unproven Hypothesis

More than eighty years ago, Otto Heinrich Warburg began his pioneering work on tumor cell metabolism, which culminated in the hypothesis that endogenous respiration defects of cancer cells, accompanied by the slow development of an enhanced ‘aerobic glycolysis’ during carcinogenesis,` would be a metabolic switch, actually defining the root of cancer [1,2]. This metabolic cancer hypothesis in its strict sense has largely been forgotten, since the era of genetic carcinogenesis concepts revolutionized science. Although changes in energy metabolism today are not regarded to be the cause of cancer, the idea of enhanced aerobic glycolysis (‘*Warburg effect*’), being a general metabolic footprint of cancer, is still present in the literature. It is even used as a rational for the clearly effective metabolic PET imaging (*^18^F-deoxy-glucose positron emission tomography*) of solid tumors, which exploits the increased accumulation of a labeled glucose-derivative in the tumor tissue, since enhanced glycolytic flux would require enhanced glucose uptake. However, the stimulation of glycolytic flux can be explained more easily by the common and often very pronounced hypoxia of solid tumors. Other molecular alterations may play an additional role for deoxy-glucose enrichment, such as increased activity of the glucose carrier Glut-1 [3,4]. Instead of using imported glucose mainly glycolytically, some tumors may convert it into fatty acids, since they contain increased amounts of fatty acid synthase [5].

The first one to criticize Warburg’s concept was Weinhouse [6], who already mentioned in the year 1956 that some normal tissues, such as brain, retina, intestinal mucosa, and kidney medulla, exhibit similar aerobic lactate production as compared with Warburg’s model, i.e. ascites tumor cells. Moreover, he stated that several tumor cells show rates of complete glucose and fatty acid oxidation, which were quite similar to normal cells. Remarkably, this topic remained a matter of debate until now. Warburg had noticed that ascites tumor cells under normoxic conditions gain roughly half of their ATP demand by fermentation of glucose to the end product lactate, while the glycolytic ATP contribution of adult mouse liver and kidney is less than 1% [7]. While the observation of a high glycolytic ATP contribution under normoxia applies also to several other tumor cells in culture, it does not apply generally to all tumor cell lines, in which both, glycolytic and oxidative ATP contributions, had been precisely measured (for review see [7]). Vice versa, some normal tissues exhibit a high contribution of aerobic glycolysis to fulfill their energy demands [7]. No statistically significant difference was observed between the groups of normal and tumor cells, if a meta-analysis of the literature was performed, which included only publications, in which both ATP generating pathways were measured in parallel.

### 1.2. Hexokinase II, a Molecular Link to the ‘Warburg Effect’

Although ‘enhanced aerobic glycolysis’ is far away from being a general and specific feature of cancer cells, molecular alterations exist in some cancers, which support a ‘*Warburg effect*’. The best studied among them is a switch in *hexokinase *(HK)* isoenzymes* during tumor development. This enzyme catalyzes the rate-limiting first step of the glycolytic pathway, the ATP-consuming phosphorylation of glucose to glucose-6-phosphate (G-6-P). Although glycolysis takes place in the cytoplasm, since 1959 some authors had described in various articles that a fraction of *hexokinase* *activity* of tumor cells appeared in a particulate cell fraction, which already pointed towards a mitochondrial association. In 1977, Bustamante and Pederson found a 20-fold increase of *hexokinase* *activity* in the azo-dye-derived rat hepatoma line H-91 as compared to normal or regenerating rat liver [8]. The enzyme was not significantly inhibited by low concentrations (< 0.6 mM) of its reaction product G-6-P in contrast to results reported earlier from *brain hexokinase*. Half of the activity was found in the mitochondrial cell fraction and the enzyme seemed to be coupled directly to the *oxidative phosphorylation* (OXPHOS), i.e. to the oxidative mitochondrial pathway of ATP synthesis.

Meanwhile, many more molecular details of this relationship have been elucidated (for review see [9]). Among the four known human isoenzymes, mainly HK II and to lesser degree HK I are upregulated in several human cancers, while the low affinity isoform HK IV is silenced [10-12]. This isoenzyme switch could be followed in detail during liver carcinogenesis. In contrast, most normal tissues express only little HK II [13, 14]. The HK II gene promoter was found to be inactivated by methylation in normal hepatocytes, while this methylation was lost in hepatoma cells during carcinogenesis [15]. The results indicated that epigenetic changes during transformation or tumor progression may underlie the isoenzyme switch. However, also a low-grade intra-chromosomal amplification of the HK II gene had been observed during hepato-carcinogenesis [16]. Both mechanisms may participate in the 100-fold increase in HK II mRNAs observed in northern blots [17-19].

A molecular basis for the suggested relationship between OXPHOS and HK II [8] was found in the discovery of a physical interaction of HK II with a pore-forming protein in the outer mitochondrial membrane, the *voltage dependent anion channel* (VDAC) [20]. ATP generated during OXPHOS in the mitochondrial matrix is transported *via *ANT *(adenosine nucleotide translocator)* through the inner mitochondrial membrane and leaves the mitochondria towards the cytoplasm *via *VDAC. In the HK II overexpressing tumor cells, ATP is, thus, directly channeled to the enzyme [21]. In this way, oxidatively generated ATP can be utilized to stimulate phosphorylation of glucose to G-6-P in the cytoplasm. The high abundance and high activity (decreased product inhibition) of the HK II *hexokinase isoform* in the tumor cells may be an important trigger of enhanced glucose utilization and lactate production of tumor cells even under normoxic conditions, under which mitochondrial ATP is available as an additional fuel of the *hexokinase reaction*. Besides these metabolic considerations, another role of HK II in creating a more malignant phenotype may be discussed, since VDAC is an essential component of the *mitochondrial permeability transition pore* (mPTP). Since the mPTP is suggested to be a major player in the intrinsic, mitochondrial pathway of apoptosis, a binding partner of VDAC may be involved in apoptosis resistance. This could be demonstrated by some authors [22, 23], while disruption of the HKII-VDAC-interaction enhanced apoptosis induction [24].

### 1.3. VHL-Gene Inactivation Mimics Hypoxia and Induces Cancer: A Second Link to Warburg

Despite induction of neoangiogenesis, hypoxia is a common phenomenon in solid tumors, to which tumor cells have to adapt their energy metabolism in order to survive and to further allow cell proliferation. This requires enhanced glucose uptake and glycolysis, since ATP cannot be delivered by the generally more efficient OXPHOS in the absence of O_2_. It is, therefore, not surprising that the expression of Glut-1 and at least one isoform of all glycolytic enzymes are increased during the HIF-1 (*hypoxia inducible factor 1*)- mediated hypoxia response. The α-subunit of the heterodimeric transcription factor HIF-1 is instable under normoxic conditions, since an O_2_-requiring hydroxylation of two prolyl residues is the prerequisite for the recognition of the α-subunit by the E3-ubiquitin ligase complex, which initiates degradation of the protein. Hypoxia thus stabilizes the protein and allows the building of sufficient amounts of active heterodimeric HIF-1, which targets many known hypoxia response genes, Glut-1 and glycolytic enzymes being among them.

Inactivation of the *von-Hippel-Lindau *(*VHL*)- tumor suppressor gene is a well-studied example of a pseudo-hypoxia reaction increasing glycolytic flux *via *HIF-1 in tumor cells [25]. The biallelic loss of this tumor suppressor gene is known to predispose patients to various benign and malignant neoplasms, mainly retinal, cerebellar and spinal hemangioblastomas, but also renal cell carcinoma, pheocromocytoma, and pancreatic cancer [26, 27]. The protein of the *VHL* gene (pVHL) is a subunit of the E3-ubiquitine ligase complex, which binds ubiquitin to all three isoforms of HIF-1α. A loss of pVHL-function therefore leads to HIF-1α stabilization, followed by a chain of events, which would normally be elicited by hypoxia. VHL inactivation by germ line mutation and *loss of heterozygosity* (LOH) is, therefore, a second, although rare, molecular alteration in tumors, which enables a ‘Warburg effect’. The glycolytic product pyruvate would normally enter the Krebs cycle *via pyruvate dehydrogenase* (PDH). Since the PDH-phosphorylating (inhibiting) enzyme *pyruvate dehydrogenase kinase 1* (PDK1) is among the HIF-1 targets, VHL-inactivation at the same time inhibits Krebs cycle and OXPHOS. As a consequence, most of the pyruvate has to be metabolized to lactate. Taken together, this represents a switch from oxidative to glycolytic ATP generation under normoxia. In the following paragraph, it will be shown that the chain of events eliciting a pseudo-hypoxia response can also have its primary origin in a mitochondrial dysfunction. The inhibition of two Krebs cycle enzymes by gene mutation can induce tumor growth, which means that enzymes of the oxidative energy metabolism act as true tumor suppressors at least in some relatively rare tumor diseases.

## 2. ENZYMES OF THE KREBS CYCLE AS TUMOR SUPPRESSORS

### 2.1. Succinate dehydrogenase: Another Mode to Mimic Hypoxia

#### 2.1.1. Familial and Non-Familial Paragangliomas

A major breakthrough in answering the question, whether mutations of mitochondrial constituents, which are involved in intermediate and energy metabolism, can be the primary cause of human tumors that was achieved in the year 2000 by Baysal and colleagues [28]. The authors analyzed the genetic basis of a relatively rare and benign inherited human tumor disease, the *hereditary paraganglioma* (PGL), for which at least three gene loci (PGL-1, -2, -3) had been suspected. Although this tumor can occur at various body sites, it is observed most often in the *carotid body* (CB), a tissue representing the major oxygen sensor in mammals. The CB stimulates the cardiopulmonary system under hypoxic conditions [29] by processes, which are thought to be governed at the cellular level by HIF-1 [29-31]. Chronic hypoxia, e.g. caused by dwelling in high altitudes or some cardiopulmonary disorders, induces cellular hyperplasia or anaplasia in the CB tissue [32-38]. Similar histologic changes observed in PGL [37, 39] first hint that some kind of HIF-1 mediated pseudo-hypoxia response may underlie PGL pathogenesis. This led to the hypothesis that the genetic defect in PGL may inactivate an essential component of the oxygen sensing machinery itself or of the signal transducing mechanisms on the way to HIF-1.

The genetic analysis of PGL-1 families [28] led to the discovery that the *SDHD* gene, encoding the small cytochrome b subunit within the mitochondrial *succinate dehydrogenase complex* (SDH) is responsible for PGL-1. Germline missense or nonsense mutations in this gene were detected together with LOH of the second allele in the tumor tissue. The *SDHD*-gene, thus, behaved like a typical tumor suppressor, since biallelic loss was necessary for tumor development. Later, it was, however, found that LOH not always occurred in the tumor tissue but some what mysteriously remained the feature of maternal imprinting, i.e. the transmission of a diseased phenotype solely through the fathers. The authors suggested that maternal imprinting may be related to the fact that in several PLG-1 families chromosomal losses (LOH) of the *SDHD* region in the tumor tissue occurred selectively in the maternal chromosome, but the reason for this remained unknown.

**Fig. (1):**
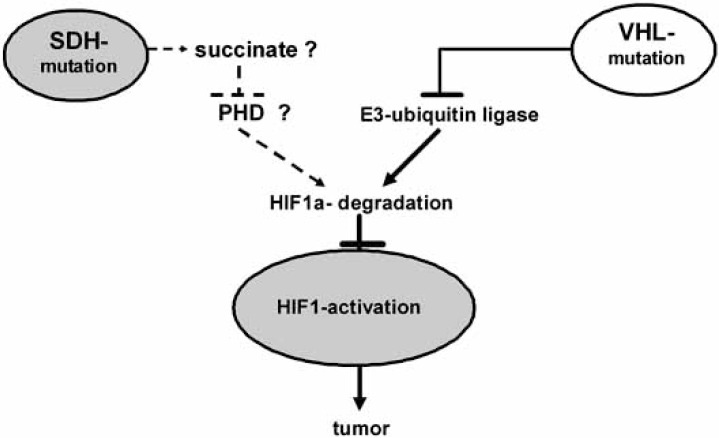
Model derived from cell culture studies to explain the ‘pseudohypoxia effect’ of succinate dehydrogenase (SDH) inactivation, which may simply inhibit prolyl hydroxylase (PHD) by succinate accumulation. PHD is required to prepare HIF1α to subsequent ubiquitin ligation and degradation. Inactivation of the well known Van-Hippel-Lindau tumor suppressor gene (VHL) directly destroys ubiquitin ligase activity.

This work demonstrated for the first time that mutations of enzymes of the oxidative mitochondrial metabolism can be tumorigenic. At this point, it was not clear as to how SDH dysfunction initiates tumor growth in the CB. SDH has a dual function in mitochondria, since it is an enzyme involved in the Krebs cycle and is part of the mitochondrial *electron transport chain* (ETC), where it is designated as ‘ETC complex II’. This means that SDH participates in the intermediary metabolism, as well as in the production of reduced flavin nucleotides, which support the electron flow, used for mitochondrial ATP synthesis. At first sight, it was not obvious as to how an inhibition of one of these two functions may lead to a pseudo-hypoxia response with HIF-1 activation, hyperplasia, anaplasia and at least development of a benign tumor. Meanwhile, a hypothesis has been deduced from cell culture studies, which proposes loss of an enzymatic function required for HIF1α-degradation as the key mediator of SDH dysfunction, thus, connecting this novel tumor suppressor with the classical *VHL* gene (Fig. **1**).

Studies using *SDHA*-mutant fibroblasts shed some light on the mechanism, although this subunit is currently not known to be prone to inactivating mutations in human tumors, while two other subunits are involved in tumorigenesis (see below). In the mutant fibroblasts, SDH-deficiency was correlated with succinate accumulation and HIF-1 nuclear translocation [40]. The latter process was not observed in ATPase-deficient fibroblasts or under cell culture conditions with enhanced production of *reactive oxygen species* (ROS). These results suggested that the accumulation of the SDH substrate succinate in the Krebs cycle may be a better candidate as a starting point of enhanced HIF-1 signaling than a decrease in mitochondrial ATP or an increase in ROS production by the defective enzyme.

Since succinate is a reaction product of the *prolyl hydroxylase* (PHD), which prepares HIF-1α to subsequent degradation, it was suggested, that product-inhibition of PHD may play the key role. This view was strongly supported by the fact that the PHD substrate, α-ketoglutarate (KG), inhibited HIF-1 activation in the mutant fibroblasts. The product/substrate equilibrium of PHD may be critically shifted also by tumorigenic SDH mutations. This may apply at least to some critical mutations of SDHB (see below), a subunit which together with SDHA forms the catalytic core, the inactivation of which should lead to succinate accumulation. In other subunits and probably depending on the location of the affected codons, enhanced ROS production may however be essential [41, 42].

In the same year, when Baysal and colleagues had published their work, the *SDHC* gene was identified as a second PGL locus. Germline mutations caused autosomal dominant paraganglioma type 3 (PGL-3), a form without genetic imprinting [43]. Shortly thereafter mutations of *SDHB* and of *SDHC* / *SDHD*, which anchor the catalytic core to the inner mitochondrial membrane, had been identified as a frequent cause of all three familial PGL-types and as a less frequent cause of non-familial cases [44].

#### 2.1.2. Pheocromocytoma and Other Tumors

Pheochromocytoma, a tumor of the adrenal medulla, which may cause secondary hypertension, occurs as a familial disease in about 10% of cases. These cases can be caused by germline mutations of various tumor suppressor genes, *VHL* being among them. This suggested that altered HIF-1 signaling may play a role in patients with familial pheochromocytoma. *SDHB* mutations were found in two of the five families screened and the same gene was occasionally affected in sporadic forms of the disease [45].

Familial *renal cell carcinomas *(RCC) are also among those tumor types, which are sometimes associated with VHL-inactivation. *SDHB* mutations were not only detected in patients suffering from a familial tumor syndrome with early onset RCC and/or PGL [46], but were found also in 4.4% of sporadic RCC cases [47]. Moreover, SDH germline mutations were observed in 13.5% of patients with Cowden’s syndrome without mutations in the tumor suppressor PTEN, which explains this rare, inherited hamartoma and tumor disease in more than 80% of cases [48].

### 2.2. Fumarate Hydratase

*Fumarate hydratase *(FH) deficiency is an encephalopathy inherited as an autosomal recessive trait, and is associated with mostly carboxyterminal germline mutations in the FH gene behind codon 250 [49-52]. Germline mutations clustering in the aminoterminal region before codon 250 were however more recently associated with an autosomal dominant tumor syndrome causing uterine and skin leimyo- mata, as well as papillary renal cell cancer [53]. In 84% of the cases, the second allele was inactivated by LOH and the FH gene, therefore, behaved as a classical tumor suppressor. The tumor suppressor concept was further underlined by the observation, that the leiomyomata showed virtually no FH enzyme activity. Occasionally, mutations and/or LOH in the chromosomal region, which contains the FH gene, were also observed in non-syndromeuous leiomyomata [54]. FH is an enzyme placed in the Krebs cycle immediately behind SDH, suggesting that tumor development may involve similar mechanisms including an important role for succinate accumulation.

## 3. OTHER NUCLEAR-ENCODED MITOCHONDRIAL PROTEINS DISCUSSED IN CANCER

### 3.1. Does Frataxin Play an Inhibiting Role in ROS-Mediated Tumorigenesis ?

Friedreich’s ataxia is a severe neurodegenerative disease of adulthood, often accompanied by cardial hypertrophy and usually leading to the patient’s death within 15 years. It is inherited as a recessive autosomal trait, caused by an intronic GAA trinucleotid expansion in the frataxin gene, which switches off transcription of the affected allele [55]. Frataxin is a mitochondrial protein, which is suggested to be involved in iron homeostasis in the mitochondria, since a reduced amount of the protein in patients with the intragenic trinucleotide expansion leads to intramitochondrial iron deposits. Although the details of frataxin function are currently a matter of debate, these deposits may indicate an insufficient transport of iron to the sites of iron-sulfur cluster biogenesis by frataxin [56-59], which in turn may hamper the incorporation of correctly iron-loaded Fe/S-clusters into various mitochondrial proteins, such as the Fe/S-containing ETC complexes. In cell cultures, frataxin inactivation was shown to result in ETC inhibition, as demonstrated by a diminished mitochondrial membrane potential, decreased O_2_-consumption and decreased oxidative ATP synthesis. Frataxin dysfunction may lead to an inhibition of mitochondrial energy metabolism. On the other hand, a dysfunction of ETC complexes, as well as deposits of free iron (by Fenton reaction) can cause enhanced generation of ROS in the mitochondrial matrix and in the intermembrane space. Oxidative stress is thought to further damage ETC complexes and other redox-sensitive proteins, such as the Krebs cycle enzyme aconitase. This oxidative stress component may further inhibit intermediary metabolism and oxidative ATP synthesis.

Ristow and colleagues analyzed for the first time a potential tumor suppressing function of frataxin in cell culture and animal models, although patients suffering from Friedreich’s ataxia are in no way prone to a higher tumor burden. The idea behind this work was a potential connection between the antioxidative properties of frataxin and ROS-mediated tumorigenesis. The authors analyzed this topic initially in murine 3T3L1 cells, which had been transfected with either a vector expressing human frataxin or a control construct [60]. Both cell clones were exposed to culture conditions with artificially enhanced ROS production. The cells overexpressing frataxin exhibited a significantly lower number of anchorage-independent foci in the culture dishes and the rate of colony formation in soft agar assays was significantly lower, indicating that frataxin protected the cells from ROS-induced transformation into a tumor phenotype. This was further supported by the observation that only cells from the anchorage-independent foci were able to induce tumor growth when xenografted to nude mice.

While oxidative stress in frataxin-deficient patients with Friedreich’s ataxia may be due to the disturbed synthesis of Fe/S-clusters and mitochondrial iron deposits, it is less obvious as to how enforced overexpression of frataxin in normal cells may protect them against ROS. The authors explained the protective effect by the observed increase in *glutathione peroxidase *(GPx) activity and in reduced thiols. GPx and the reduced form of glutathione play an important role in the detoxification of H_2_O_2_, which is built as a product of the SOD- *(superoxide dismutase*) reaction. *Superoxide dismutase 2 *(SOD2) is a mitochondrial enzyme, which detoxifies superoxide radicals, released mainly from ETC complexes I and III into the mitochondrial matrix.

In a next step, the authors investigated the potential tumor suppressing effect of enforced frataxin expression in the colon cancer lines MIP101, DLD2 and HT29, which lack endogenous expression of the protein. Mitochondrial oxidative metabolism was enhanced in the transfected cells, as could be shown by an increase of mitochondrial membrane potential, cellular respiration and ATP content, as well as aconitase activity. Increased aconitase activity may be explained in part by decreased oxidative stress. Again, the frataxin cells exhibited a lower colony formation rate in soft agar essays and a lower rate of tumor growth after xenotransplantation to nude mice [61]. The most direct evidence for a role of frataxin in carcinogenesis was reported by the same group using targeted hepatic disruption of frataxin expression in mice. The animals had reduced life spans and developed multiple hepatic tumors, in which high apoptotic and mitotic (Ki-67) indices were observed [62]. The liver specimen showed elevated levels of *thiobarbituric-acid reactive substances *(TBARS), a marker of lipid peroxidation, and elevated levels of oxidized glutathione, a classical oxidative stress marker. Activities of those mitochondrial enzymes, which contain Fe/S-moieties, i.e. aconitase and ETC complexes I, II, III, were reduced. In accordance with an inhibited oxidative metabolism, the ATP content of livers from knock-out animals was decreased.

The authors did not develop a detailed hypothesis regarding the mechanism of liver tumorigenesis, but speculated that an observed reduction in p38-MAP-kinase phosphorylation may play a role, since activation of this type of *mitogen activated protein kinase* had been discussed earlier as a factor suppressing the formation of hepatic tumors [63, 64]. Although the authors did not discuss this issue, it might be speculated, whether the observed inhibition of SDH (complex II) may participate in tumorigenesis.

### 3.2. Other Genes Related to Oxidative Stress

SOD2 is the main enzyme to detoxify the superoxide generated by the ETC. Since probably all of the superoxide generated by ETC complex I and a part of that produced by complex III is released into the mitochondrial matrix, SOD2 is located in this compartment. Import of the monomeric precursor protein requires a translocation through both mitochondrial membranes, mediated by the *translocators of the outer *(TOM) *and inner membrane *(TIM), for which a mitochondrial targeting sequence at the aminoterminus is needed. A Val/Ala polymorphism in codon 16 of this leader peptide is known, which predicts a profound conformational change between α-helix and β-sheet. Ala-16 homozygosity has been shown to be associated with poorer transport efficiacy, leading to a dicreased intramitochondrial formation of the homotetrameric mature enzyme, which in turn reduces the antioxidative capacity of mitochondria due to a lower enzyme activity [65]. The lowered SOD2 activity may usually be without major consequences for the cell, but may lead to oxidative stress, when other antioxidative mechanisms are disturbed at the same time. Li and colleagues observed that low blood selenium levels enhance the prostate cancer risk in individuals homozygous for the Ala-16 leader sequence [66]. This observation may be interpreted in terms of a synergism between decreased SOD2-activity and lowered activity of *glutathione peroxidase *(GPx), a selenium-dependent enzyme.

Selenoprotein P is the most important selenium binding protein, which accounts for at least 40% of total plasma selenium load [67]. The protein contains a polymorphism in codon 234, the alanine allele being associated with lower plasma selenium concentrations in men, which affect GPx activity [68]. It was therefore a logical step to investigate a possible synergism between Ala234 and the SOD2-leader polymorphism. Indeed, synergistic effects were observed, since men homozygous for the ‘wrong allele’ of both genes had a significantly enhanced risk of prostate cancer (*odds ratio *(OR) 1.43) or clinically aggressive prostate cancer (OR 1.60). The OR became even more pronounced in men, who were strong smokers [69].

### 3.3. Mutations Causing ETC Defects by Generating a Fragile Mitochondrial Genome

The precursors of most mitochondrial proteins are encoded by nuclear genes, translated into polypeptides at cytoplasmic ribosomes and imported into the mitochondria, to which they are targeted by mitochondrial leader sequences at their amino terminus. This rule applies also to the vast majority of polypeptides, which constitute the five OXPHOS complexes of the inner mitochondrial membrane, i.e. ETC complexes I to IV and the ATP-synthetase (F_0_/F_1_-ATPase, complex V). However, with the exception of complex II (succinate dehydrogenase), all OXPHOS complexes contain at least one polypeptide, which is encoded in a small (16.6 kb) circular and intron-free DNA molecule in the mitochondrial matrix space, the mtDNA. Altogether the mtDNA encodes 13 OXPHOS polypeptides, as well as 2 ribosomal and 22 tRNAs needed for their translation by ribosomes of the mitochondrial matrix.

*DNA polymerase γ *(POLG) is the enzyme, which replicates this mitochondrial genome. It is necessary to maintain a functional ETC and OXPHOS. Interestingly, heterozygeous missense-, nonsense- or frameshift-mutations in the POLG gene do usually not cause a depletion of mtDNA, but lead to a fragility of the mitochondrial genome, which is prone to multiple large scale deletions in a proportion of mtDNA molecules, which can be detected in Southern blots of skeletal muscle DNA. These deletions usually affect some mtDNA-encoded tRNA genes besides protein-coding sequences and can inhibit ETC complexes I, III, and IV, as well as the ATP synthetase. The functional defect is translated into a phenotype, which predominantly affects skeletal muscles (muscle weakness and external ophthalmoplegia) and is usually transmitted as an autosomal dominant trait, designated adPEO (*autosomal dominant progressive external ophthalmoplegia*) [70].

The POLG gene contains a polymorphic CAG repeat [71, 72], which is conserved in all species investigated so far. Since the clearly dominating repeat length in the human population is 10, selective pressure seems to occur to conserve this length [73]. Men with homozygous deviations from this dominating repeat length were recently shown to have an increased risk of developing testicular germ cell cancer, mainly seminomas [74]. No correlation of repeat length with other clinico-pathological features was observed. Although the fraction of persons with a homozygous ‘non-10’ allele was significantly higher among cancer patients (4,9%) as compared to the general population (1.3%), the generally low frequency shows that the aberrant repeat length can be viewed only as mild risk factor for tumor development.

## 4. ROLE OF MTDNA-ENCODED MITOCHONDRIAL PROTEINS IN CANCER

### 4.1. ATPAse Mutation Enhancing Tumorigenicity of Prostate Cancer Cells in Nude Mice

Since more than ten years, a growing number of articles described somatic mutations of the mitochondrial genome (mtDNA) in human tumors, by comparing tumor and adjacent normal tissue or blood usually with sequencing techniques. Sometimes more sophisticated techniques were used, dedicated to the detection of a low burden of mutated mtDNA (heteroplasmy). Often these mutations occurred in the non-coding, so called D-loop region, or were silent base substitutions. Usually, these base exchanges did not include pathogenic mutations known from ‘mitochondrial disorders’ and could not be predicted to have functional consequences by other criteria, such as introduction of stop-codons, frame-shifts or alteration of conserved tRNA loop-structures. A large proportion of these mutations actually corresponded to known human polymorphisms. They could be suspected to be caused by oxidative stress and to be enriched in the tumor tissue as a consequence of the clonal expansion of a single somatic cell.

In the year 2005, however, Petros and colleagues demonstrated a direct causal connection between a known pathogenic mtDNA mutation (T8993G in the ATPase-6 gene) and the tumorigenecity of PC3 prostate cancer cells in nude mice [75]. This mutation was well known to elicit mitochondrial disorders. It can be responsible either for a severe CNS disease leading to symmetrical necroses in the brain and early death (Leigh syndrome) or to the less severe disease NARP *(neuropathy, ataxia and retinitis pigmentosa)*, which seems to depend on the exact proportion of mutant and normal DNA in the CNS. Generally, very high burdens of the mutation (> 95% of total mtDNA in the relevant tissues) are thought to be necessary to cause any mitochondrial disorder. This suggests that the ATP synthetase function is significantly hampered only by extremely high mutation loads, but even lower percentages of mutant DNA may cause enhanced ROS production. Petros and colleagues detected this transversion as a heteroplasmic germline mutation in a patient with prostate cancer. The authors introduced mtDNA carrying the mutation and in parallel unaffected mtDNA from the same patient into mtDNA-depleted PC3 prostate cancer cells, using the cybrid technique [76]. The PC3 cells with the mitochondrial genotype 8993G developed tumors with a significantly enhanced growth rate in nude mice, as compared to the wild-type. Effects of other variations in the mitochondrial or nuclear genomes were excluded by this sophisticated approach. The mitochondrial haplogroup, as well as other mtDNA positions potentially influencing OXPHOS were identical in both xenografted tumor cell lines (genotype of the patient). The nuclear background was that one of PC3 tumor cells in both cases. Therefore, the growth difference between the two manipulated tumor cell clones (‘transmitochondrial cybrids’) could be attributed to the T8993G mutation. Although unproven in their article, the authors discussed a probable role of enhanced mitochondrial ROS production by the mutant cells to support tumor growth, since ROS are known to influence transcription factors [77], signal transducing kinases and regulatory phosphatases and angiogenic factors. The ATP synthetase mutation T8993G is known to induce enhanced ROS production in other cell types, including cybrids based on the osteosarkoma cell line 143B. Increased, but still moderate levels of mitochondrial ROS production may be the cause of enhanced tumorigenecity, as suggested by the authors.

### 4.2. Spectrum of Human Tumor Diseases, in which mtDNA Mutations were Observed

Despite Warburg’s early suggestion of a causal role of OXPHOS defects in cancer development [1, 2], the mitochondrial genome of cancer cells had not been studied until 1998, when Polyak and colleagues sequenced the mtDNA of ten human colorectal cancer cell lines [78]. The authors detected 12 mutations in 7 of the cell lines (70%). Most base exchanges occurred in rRNA genes or were missense mutations, only two were functional without any doubt (frame shift and chain termination). Although tumor cells often contain a relatively small number of mitochondria with a few mtDNA molecules per mitochondrion, the resulting number of mtDNA copies per cell still guarantees, that a new somatic mtDNA mutation initially represents only a minor fraction of total tumor mtDNA, a state called heteroplasmy. Having this situation in mind, it was rather surprising that most of the mutations were found to be homoplasmic, at least at the sensitivity level of DNA sequencing. Homoplasmic mtDNA mutations were later often observed in human tumor tissues by other authors and do not represent a rare event or a special feature of long cultivated cell lines. As an obvious explanation for homoplasmy, Polyak and colleagues suggested that the mutated mtDNA was dominant at the intracellular and intercellular level in the tumors, from which the cell lines had been derived. This was an important suggestion, since it implied that at least one of the observed mutations per mitochondrial genome should have conferred a selective advantage and, thus, be ‘functional’. This hypothesis would suggest a role of mtDNA mutations to promote the proliferation of certain types of tumor cells just in the sense of Warburg’s hypothesis. However, selective pressure is not the only explanation for homoplasmy, and not even the simplest one, as will be outlined below (see section 4.3).

In the same year, Habano and colleagues analyzed 45 colorectal tumor samples for ‘microsatellite instability’ within some homopolymeric sequence tracts of mtDNA [79]. The term ‘microsatellite instability’ was misleading in a double sense. These sequence tracts do not fullfill the definition of microsatellites. In addition, the low mismatch repair activity in mitochondria is not the only explanation for their length variability in human tumors, as discussed below (see section 4.3). The term was probably coined to remind nuclear microsatellite instability due to mismatch repair defects in inherited non-polyposis colorectal cancers. The authors detected length changes of the polycytosine tract of the hypervariable region 2 (HVR2) of the mitochondrial D-loop in 20 tumors (44%). This sequence tract, often referred to as D310 in the tumor literature, is meanwhile known to be highly variable in several solid tumor types [80]. It is non-coding (like the whole D-loop) and two different length variants are known to be the dominating constitutive genotypes in normal human tissues, often in a heteroplasmic mixture. Nevertheless, the tract is essential for binding and correct nuclease processing of a RNA primer, which is required for mtDNA replication. In tumors, rare length variants are more frequently observed and even rather long tracts occur, which are never seen as constitutive genotypes in blood samples. In the cancer samples originally analyzed by Habano and colleagues, the authors later described some complex I mutations in 16% of cases in a more detailed analysis [81].

Parrella *et al. *[82] investigated 18 primary breast cancers (invasive ductal carcinomas) from 17 women by direct sequencing. MtDNA mutations were detected in 11 tumors (61%), 42% being length alterations in D310. The remaining were base substitutions in several protein-coding genes. Mutations in the coding and non-coding regions of the mitochondrial genome were detected in half of the investigated head and neck cancers [83, 84], in 64% of bladder and 43% of lung cancer patients [84]. Among patients with multicentric hepatocellular carcinoma, 68% exhibited mtDNA mutations at least in one of the tumors, including a high frequency of D310 length variations (62% of patients).

We and other authors detected a high frequency of D-loop mutations in astrocytic brain tumors [85-87]. We applied D310 length variation to verify clonality in gliomatosis cerebri, an astrocytic tumor disease, in which several brain lobes are diffusely infiltrated without clearly visible tumor margins [88]. The loss of a heteroplasmic D310 genotype in one hemisphere affected by gliomatosis corresponded with a p53 mutation in this hemisphere, thus, verifying the clonal expansion of the altered mitochondrial genotype during tumor growth. By screening the mitochondrial genome with time consuming temperature gradient gel electrophoresis, followed by sequencing, mutations were identified in 40% of medulloblastomas, the most frequent brain tumor of childhood [89].

Studies focusing on the highly susceptible D-loop region or sequencing a few mitochondrial genes in addition, tended to find a lower proportion of tumors, harboring a base exchange or insertions/deletions as compared to studies, which tried to assess the mitochondrial genome as much as possible. A recent chip analysis of 94% of the mitochondrial genome in 15 pancreatic cancers detected at least one somatic mutation in every tumor, suggesting that indeed most solid tumors contain mtDNA mutations.

### 4.3. Is There any Benefit for the Tumors? Positive Selection Versus Random Drift

Even polymorphisms of the mtDNA observed in healthy mammals are not necessarily neutral, e.g. during the development and aging of organs. This aspect of mitochondrial genetics had already been illuminated in a study creating heteroplasmic mice by genetic engineering [90] in the year 1997. The mitochondrial genome of two normal healthy mouse strains had been mixed to achieve a balanced artificial heteroplasmy. The two genomes could easily be distinguished by means of PCR. The authors even analyzed heteroplasmic mice with two different nuclear genomes to exclude a potential role of nuclear background. The unexpected result of that study was a tissue-specific up- or downregulation of one of the two mitochondrial genomes during development and adult life of the mice. This result demonstrated that even mitochondrial genomes, which do not carry any known functional mutation, can differ in their replicative fitness in different organs of mammals.

Originally, the surprising observation of homoplasmy for a significant proportion of somatic mtDNA mutations in human tumor cells was also interpreted as a replicative advantage of mutated mtDNA and/or a growth or survival advantage for the cells carrying the affected mitochondria [78, 84, 91]. Having in mind the above cited results in heteroplasmic mice, even a proportion of the D-loop, rRNA, tRNA or missense mutations in non-conserved codons may be interpreted to confer a ‘selective advantage’.

However, it is not possible to draw this conclusion simply from the observed frequency of homoplasmic tumors, as could be demonstrated by computer modeling. Coller and colleagues [92] have shown that a sufficient random drift of heteroplasmic mtDNAs theoretically occurs even under conditions of unbiased mtDNA replication and sorting during cell divisions, which can explain the observed frequency of homoplasmy. The computer models were based on some assumptions regarding mtDNA content, mutation rate and number of cell divisions minimally required to generate a detectable solid tumor, which can not be proven in detail. However, even rather conservative estimations of these parameters yielded results, which were in good accordance with the observed rate of homoplasmy. This study forced investigators to accept the notion that homoplasmy of a tumor mutation is no proof of functional relevance. It seems, thus, likely that a large proportion of all observed mutations are nothing else but indicators of a clonal cell expansion, which may however be exploited as a tool of early cancer detection anyway.

Some sentences may still be dedicated to the usage of the word ‘somatic mutation’ in the tumor literature, which implies that in all cases a base exchange or deletion/insertion occurred *de novo* in a tumor cell or precursor cell. This assumption seems questionable, at least for some nucleotide positions of mtDNA, which are often heteroplasmic in normal human tissues. The poly-cytosine tract D310, a so called ‘hot spot of somatic mutations’ in solid tumors, is a good example. The tract occurs in the blood of roughly half of all healthy individuals in one of the two variants C_7_TC_6_ or C_8_TC_6_, as determined by sequencing. Especially individuals with the longer tract are usually heteroplasmic, i.e. the lymphocytes (and other tissues) contain lower amounts of the shorter variant, as detected by PCR techniques. Even other variants of the tract can occur in normal tissues. It seems, thus, reasonable to assume that length alterations of this polycytosine stretch in solid tumors, observed during sequencing, are often caused simply by relative shifts between two or more pre-existing heteroplasmic genotypes of the precursor cell. In our laboratory, mtDNA from some glioblastomas had been cloned for extensive analysis of low abundant mtDNA mutations. The ‘constitutive genotype’ of one patient in this study (normal brain tissue) consisted of two mitochondrial genomes, which could be distinguished by D310 length and two D-loop transitions. One of these genomes was clearly lost in the glioblastoma [85]. This change was attributed to heteroplasmic drift, since three independent events would have been necessary to explain this result by somatic mutations. Unless investigated by specific PCR-assays for a given nucleotide position, one can never be sure that adjacent normal tissue did not contain low amounts of the mtDNA observed as the dominant genotype in a tumor. However, the exact nature of the observed sequence alterations in human tumors plays no role for their potential use in cancer detection, as long as they represent clonal cell expansions. In the following sections, the term ‘mutation’ will be used regardless of the problems associated with it especially regarding D310.

### 4.4. Are mtDNA Mutations Associated with Patient Survival?

**Fig. (2):**
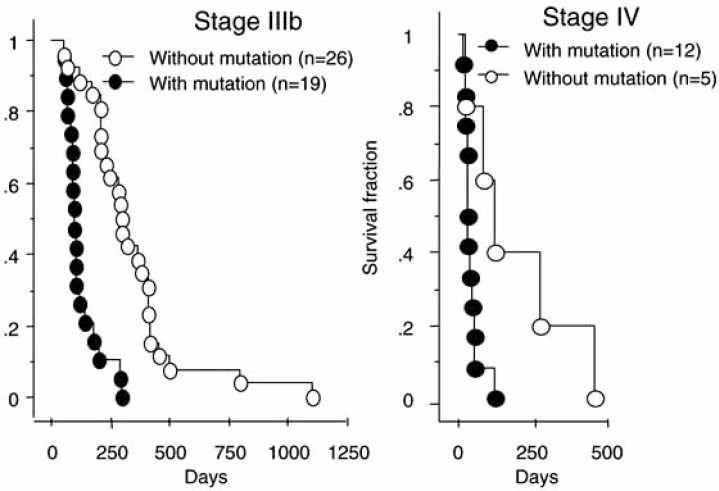
Comparison of the cumulative survival rates between NSCLC patients with and without mitochondrial DNA mutations (modified according to [93]).

At least two studies reported preliminary data, suggesting that mutated mtDNA is a marker of poor survival in advanced cancer patients. In a large series of 202 patients suffering from NSCLC (*non small cell lung cancer*) of various histological subtypes, mitochondrial rRNA genes and D-loop were analyzed. Mutations were found only in the latter region in 34.7 % of cases [93]. No significant differences in mutation frequency occurred between the histological subtypes (adenocarcinoma, squamous cell carcinoma, bronchoalveolar carcinoma, large cell carcinoma), but mutations accumulated significantly in tumor grades IIIb or higher (p<0.0001). Tumor grades had been determined according to the international staging system [94]. The most important result of that study was the significantly decreased survival of advanced cancer patients harboring mtDNA mutations. This difference was observed within grade IIIb and within grade IV (Fig. **2**). Although a prospective study (fresh material collected prior to cancer treatment), the article did not contain any information regarding chemo- and radiotherapy.

**Fig. (3):**
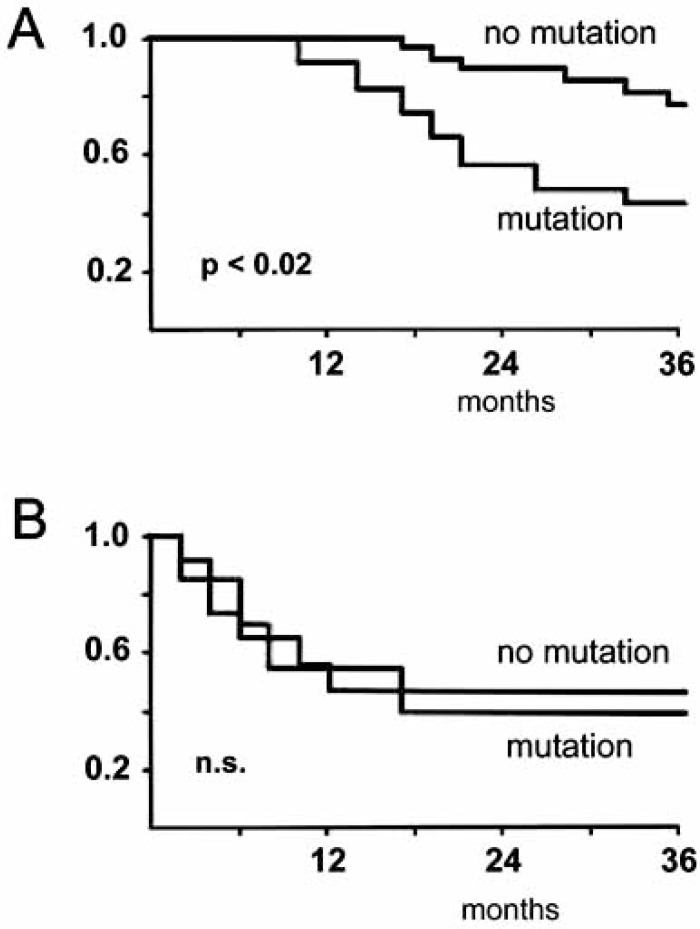
Comparison of cumulative survival rates in patients with stage III colorectal cancer with and without D-loop mutations, either receiving fluorouracil-based adjuvant chemotherapy (A) or receiving no adjuvant chemotherapy (B). Modified according to [95].

Lievre and colleagues [95] retrospectively analyzed the D-loop region of 365 colorectal cancer patients from the Digestive Cancer Registry of Cote d’Or (France). Mutations were detected in 38.3% of the samples. The 3-year survival rate of these patients was 53.5% as compared to 62.1% in the negative cases (p = 0.05). The benefit of a non-mutated status became more pronounced in advanced cancers (Fig. **3**). If all stage III cases were included, the 3-year survival rates of the groups with and without mutation were 47.4% versus 70.0%, respectively (p<0.02). If fluorouracil had been used as adjuvant chemotherapy, the difference was even more pronounced, i.e. 45.4% *vs*. 78.3% (p<0.02).

Although the results of both studies suggest mtDNA as an additional prognostic marker to refine histology-based prognoses, the suitability of such approaches remains questionable, unless the treatment regimens (chemotherapy and radiation) are more precisely reported and controlled in such analyses. This is not a simple issue in retrospective studies, but could be done by collecting mtDNA from patients, who are dedicated to participate in controlled clinical trials performed for testing new therapy regimens.

## 5. mtDNA AS A POTENTIAL TOOL OF EARLY CANCER DETECTION

### 5.1. Are mtDNA Mutations Suitable for Early Cancer Detection?

Although the frequency of mtDNA mutations in solid human tumors is still a matter of debate with sometimes large differences occurring between studies, several studies suggested that D310 length variation and base substitutions in the whole mitochondrial genome are often already detectable in a premalignant stage. In patients with a clinically manifested cancer, these mtDNA alterations can often be detected in paired bodily fluids, probably allowing the development of non-invasive diagnostic assays. Bodily fluids have been analyzed for the first time ten years ago by Fliss and colleagues in cancers of head and neck (n = 13), bladder (n = 14) and lung (n = 14) [84]. In patients harboring mutated mtDNA, in their bladder carcinoma, sequence variants were generally detectable in the paired urine samples. Most paired saliva samples of head and neck cancers were informative too, while the analysis of bronchoalveolar lavage (BAL) of lung cancers was difficult due to the dilution of tumor cell DNA in the relatively large amounts of BAL-fluid. Highly sensitive oligonucleotide mismatch ligation assays, created to specifically amplify a single base exchange, were successful in two cases, thus, delivering at least the proof of principle. Since mtDNA usually exhibits a much higher copy number per cell as compared to nuclear single copy genes, this abundance may facilitate PCR-detection in diluted samples. This is well known to the forensic community, since mtDNA can sometimes be used to analyze DNA-traces, for which commonly used markers fail. In the bodily fluids of cancer patients, a second problem occurs, i.e. the admixture of tumor cell DNA with DNA of normal epithelial cells, which may also be released into the fluids analyzed. This may be regarded as a severe problem, since it hampers all sequencing strategies, including modern chip technologies (see below), which are not extremely sensitive in discovering heteroplasmic base positions.

Fliss and colleagues, thus, measured the relative abundance of mutated mtDNA as compared to mutated p53 nuclear DNA in two BAL samples and found the mtDNA sequence to be 19 and 220-fold more abundant. These results suggested that mtDNA may be a more sensitive tool for early cancer detection as compared with nuclear genes. Later, the same group identified mtDNA mutations of three prostate cancer patients in paired urine and, more surprisingly, plasma samles. In one case, the alteration was present in the corresponding PIN lesion (*prostate intraepithelial neoplasm*).

In a large series of 137 premalignant lesions of the head and neck from 93 patients, analyzed selectively for D310 status, 22% of the benign hyperplasias, but 62% of squamous carcinomas *in situ*, harbored an altered length of the polycytosine tract [96]. The same marker was analyzed in ductal lavage (DL) of the breast in a small series of 14 women with no clinical evidence of breast cancer [97]. Nine of the women were at high-risk of developing cancer, since they harbored germline mutations of the *BRCA1* (*breast cancer* *1*) gene, while five women were known to be non-carriers. Only three (30%) of the carriers exhibited detectable length alterations of D310 in DL, which seems to occur in about 30% of breast cancer tissues [80].

### 5.2. mitoChip-Technology

The small control-region of mtDNA replication and transcription is usually designated in its total length as ‘D-loop’ in the tumor literature, although the term *displacement loop* (D-loop) originally characterized only a portion of the control region, able to built a loop structure by binding a short single-stranded primer, displacing the two strands of mtDNA. Although the control region seems to harbor the highest density of point mutations in human tumors, the majority of mutations may be missed be focusing on this region, which comprises only about 6.5% of total mtDNA. Since conventional capillary-sequencing of the complete mitochondrial genome (16.6 kb) is laborious and time consuming, parallel sequencing strategies had to be developed. Hybridization of fluorescent DNA samples to oligonucleotide arrays offers the opportunity to sequence the whole mitochondrial genome in a single hybridization experiment. Such arrays (MitoChip) have been constructed on the basis of the Custom Resequencing Microarray platform from Affymetrix (USA). The chip contains around 300.000 regions of ~500 µm^2^ (features), each representing an oligonucleotide of 25 bp. Each mtDNA position is represented by four features, which differ only by base #13, being one of the four DNA nucleotides. Fluorescent mtDNA samples, generated by long-PCR and fragmentation, will hybridize most efficiently to the feature representing an exact match, which will thus show the brightest fluorescence. The position of this feature is recognized by a microarray-scanner. This technique showed a nearly perfect reproducibility in repeated hybridizations (>99.99%) and was able to detect mutations in six of the nine bodily fluid samples (urine, pancreatic juice) from patients with bladder cancer (n = 5) or pancreatic cancer (n = 4) [98]. While the D-loop was omitted in the first generation chip due to high CG-content, this important region was included in the second version (v2.0), which moreover contained a redundant tiling for 500 of the most common haplotypes [99]. Thus, MitoCHip (v2.0) allowed for the first time the examination of the whole mitochondrial genome in a single hybridization experiment.

Sui and colleagues [100] analyzed 14 precancerous lesions of the digestive tract, all of which were found to exhibit at least one mutation. Seven of these samples had been obtained by endoscopic biopsies from Barrett esophagus, showing Barret mucose without concomitant dysplasia (n = 2), low-grade dysplasia (n = 4) or high-grade-dysplasia (n =1), thus, demonstrating mtDNA alterations even in the absence of histopathologic evidence of dysplasia. The remaining samples analyzed had been taken from four patients with colorectal adenomas (including one sessile serrated adenoma) and from three patients undergoing colectomy for long-standing idiopathic bowel disease with concomitant dysplasia. None of the analyzed adenomas had shown histopathologic evidence of invasive adenocarcinoma.

**Fig. (4):**
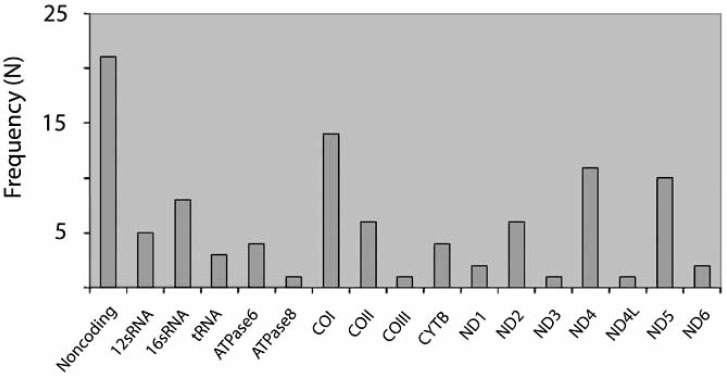
Distribution of mtDNA mutations ovserved with the MitoChip technology in preneoplastic lesions of the gastrointestinal tract (modified according to [100]). Abbreviations = mitochondrial genes according to international notation (see www.MITOMAP.com).

The affected sites of mtDNA were distributed over the whole mitochondrial genome (Fig. **4**), again demonstrating the necessity of a global approach. The majority of alterations were heteroplasmic.

Still using the initial MitoChip version (v1.0), supplemented by capillary sequencing of the D-loop, Jakupciak *et al. *recently analyzed a series of early stage cancers, including bladder (n = 3), kidney (n = 13) and lung (n = 8) and paired bodily fluids, urine or BAL respectively [101]. Mutations of the mtDNA, heteroplasmic in most cases, were detected in all three bladder cancers. They were found in 69% of kidney and 79% of lung cancers. The paired bodily fluids exhibited mutations, i.e. their genotype was distinct from the constitutive genotype in blood, in 36% of bodily fluid samples, including 43% of BAL samples.

## 6. CONCLUDING REMARKS

Several mitochondrial and nuclear genes of mitochondrial components have been associated with cancer. At least for some nuclear genes, which code polypeptides of enzymes involved in the Krebs cycle, a relationship between gene mutation and cancer development in humans is clearly proven. These genes play a role mainly in rare inherited tumors, but screening for somatic mutations is of interest in some sporadic tumor types too.

Despite their high frequency in most solid human tumor types investigated so far, a causal role for a significant portion of mtDNA mutations in cancer cannot be claimed. Although some mtDNA mutations may enhance tumor growth, the enrichment of mutated mtDNA in solid human tumors can be explained by random drift, without need of any selective advantage. Irrespective of their biological role, mtDNA mutations may be a tool for early cancer detection. Recent studies, using the MitoChip technology, suggest that heteroplasmic and homoplasmic base exchanges are detectable in the majority of solid human tumors, even in early tumor stages or precancerous lesions. Especially in tumors seeding cells into an easily accessable bodily fluid, the screening of such fluids may be a hopeful technique. The MitoChip technology has to be tested in larger series of early cancers to allow an estimation of its practical suitability. However, already at this point two major issues have to be discussed.

First of all, one has to evaluate, what is a meaningful definition of a ‘mutation’, indicative of cancer growth. It is likely that global sequencing approaches will sometimes detect sequence differences between bodily fluid and blood, which are not observed in the tumor tissue of the same patient, e.g. in the recent study of Jakupciak and colleagues, this situation occurred in five patients [101]. Several explanations can be proposed, including oxidative stress in normal epithelial tissues, pre-existing tissue-specific heteroplasmy, or technical reasons. The observed discrepancies between tumor tissues and corresponding fluids imply that global mutation data must be interpreted with care. At least, it will be necessary to learn more about the frequency of mismatches between blood and bodily fluids in unselected individuals without known cancer, premalignant lesions or inflammatory disease in the organ of interest.

A second point requiring discussion is a technical one. As already stated by Sui *et al*. [100], it is important to stress that chip based chemistry is best suited for detection of single base pair alterations, but it is likely that many insertions/deletions, frameshift mutations, and large scale deletions are missed by this technique. Generally, array technology should be much less sensitive for detection of heteroplasmic mutations in a huge excess of wild-type mtDNA, e.g. one can speculate that early detection of recurrence of a bladder or colon carcinoma, which contains a previously sequenced point mutation (follow-up), should be more meaningful by exploiting specific PCR-assays. However, if the goal is development of techniques for early detection of previously unknown cancers (screening), the whole mitochondrial genome must be analyzed. This can be done efficiently only by array technology, which has already delivered the proof of principal for this purpose. The development of this technology is, thus, connected with great expectations for the future.

## References

[1] Warburg O (1956). On respiratory impairment in cancer cells. Science.

[2] Warburg O (1956). On the origin of cancer cells. Science.

[3] Wachsberger PR,  Gressen EL,  Bhala A,  Bobyock SB,  Storck C,  Coss RA,  Berd D, Leeper DB (2002). Variability in glucose transporter-1 levels and hexokinase activity in human melanoma. Melanoma Res.

[4] Chang S,  Lee S,  Lee C,  Kim JI,  Kim Y (2000). Expression of the human erythrocyte glucose transporter in transitional cell carcinoma of the bladder. Urology.

[5] Thupari JN,  Pinn ML,  Kuhajda FP (2001). Fatty acid synthase inhibition in human breast cancer cells leads to malonyl-CoA-induced inhibition of fatty acid oxidation and cytotoxicity. Biochem. Biophys. Res. Commun.

[6] Weinhouse S (1956). On respiratory impairment in cancer cells. Science.

[7] Zu XL,  Guppy M (2004). Cancer metabolism: facts, fantasy, and fiction. Biochem. Biophys. Res. Commun.

[8] Bustamante E,  Pedersen PL (1977). High aerobic glycolysis of rat hepatoma cells in culture: role of mitochondrial hexokinase. Proc. Natl. Acad. Sci. USA.

[9] Mathupala SP,  Ko YH,  Pedersen PL (2006). Hexokinase II: cancer's double-edged sword acting as both facilitator and gate-keeper of malignancy when bound to mitochondria. Oncogene.

[10] Rempel A,  Bannasch P,  Mayer D (1994). Differences in expression and intracellular distribution of hexokinase isoenzymes in rat liver cells of different transformation stages. Biochim. Biophys. Acta.

[11] Mayer D,  Klimek F,  Rempel A,  Bannasch P (1997). Hexokinase expression in liver preneoplasia and neoplasia. Biochem. Soc. Trans.

[12] Pedersen PL,  Mathupala S,  Rempel A,  Geschwind JF,  Ko YH (2002). Mitochondrial bound type II hexokinase: a key player in the growth and survival of many cancers and an ideal prospect for therapeutic intervention. Biochim. Biophys. Acta.

[13] Wilson JE (1995). Hexokinases. Rev. Physiol. Biochem. Pharmacol.

[14] Wilson JE (2003). Isozymes of mammalian hexokinase: structure, sub-cellular localization and metabolic function. J. Exp. Biol.

[15] Goel A,  Mathupala SP,  Pedersen PL (2003). Glucose metabolism in cancer Evidence that demethylation events play a role in activating type II hexokinase gene expression. J. Biol. Chem.

[16] Rempel A,  Mathupala SP,  Griffin CA,  Hawkins AL,  Pedersen PL (1996). Glucose catabolism in cancer cells: amplification of the gene encoding type II hexokinase. Cancer Res.

[17] Johansson T,  Berrez JM,  Nelson BD (1985). Evidence that transcription of the hexokinase gene is increased in a rapidly growing rat hepatoma. Biochem. Biophys. Res. Commun.

[18] Rempel A,  Bannasch P,  Mayer D (1994). Microheterogeneity of cytosolic and membrane-bound hexokinase II in Morris hepatoma 3924A. Biochem. J.

[19] Mathupala SP,  Rempel A,  Pedersen PL (1995). Glucose catabolism in cancer cells. Isolation, sequence, and activity of the promoter for type II hexokinase. J. Biol. Chem.

[20] Nakashima RA,  Mangan PS,  Colombini M,  Pedersen PL (1986). Hexokinase receptor complex in hepatoma mitochondria: evidence from N,N'-dicyclohexylcarbodiimide-labeling studies for the involvement of the pore-forming protein VDAC. Biochemistry.

[21] Arora KK,  Pedersen PL (1988). Functional significance of mitochondrial bound hexokinase in tumor cell metabolism. Evidence for preferential phosphorylation of glucose by intramitochondrially generated ATP. J. Biol. Chem.

[22] Pastorino JG,  Hoek JB (2003). Hexokinase II: the integration of energy metabolism and control of apoptosis. Curr. Med. Chem.

[23] Vyssokikh MY,  Brdiczka D (2003). The function of complexes between the outer mitochondrial membrane pore (VDAC) and the adenine nucleotide translocase in regulation of energy metabolism and apoptosis. Acta Biochim. Pol.

[24] Zaid H,  Abu-Hamad S,  Israelson A,  Nathan I,  Shoshan-Barmatz V (2005). The voltage-dependent anion channel-1 modulates apoptotic cell death. Cell Death Differ.

[25] Godinot C,  de Laplanche E,  Hervouet E,  Simonnet H (2007). Actuality of Warburg's views in our understanding of renal cancer metabolism. J. Bioenerg. Biomembr.

[26] Neumann HP,  Wiestler OD (1991). Clustering of features of von Hippel-Lindau syndrome: evidence for a complex genetic locus. Lancet.

[27] Brauch H,  Kishida T,  Glavac D,  Chen F,  Pausch F,  Hofler H,  Latif F,  Lerman MI,  Zbar B,  Neumann HP (1995). Von Hippel-Lindau (VHL) disease with pheochromocytoma in the Black Forest region of Germany: evidence for a founder effect. Hum. Genet.

[28] Baysal BE,  Ferrell RE,  Willett-Brozick JE,  Lawrence EC,  Myssiorek D,  Bosch A,  van der Mey A,  Taschner PE,  Rubinstein WS,  Myers E.N  Richard, C.W. 3rd, Cornelisse CJ,  Devilee P,  Devlin B (2000). Mutations in SDHD, a mitochondrial complex II gene, in hereditary paraganglioma. Science.

[29] Gonzalez C,  Almaraz L,  Obeso A,  Rigual R (1994). Carotid body chemoreceptors: from natural stimuli to sensory discharges. Physiol. Rev.

[30] Semenza GL (1999). Perspectives on oxygen sensing. Cell.

[31] Bunn HF,  Poyton RO (1996). Oxygen sensing and molecular adaptation to hypoxia. Physiol. Rev.

[32] Arias-Stella J,  Bustos F (1976). Chronic hypoxia and chemodectomas in bovines at high altitudes. Arch Pathol. Lab. Med.

[33] Arias-Stella J,  Valcarcel J (1976). Chief cell hyperplasia in the human carotid body at high altitudes; physiologic and pathologic significance. Hum. Pathol.

[34] Edwards C,  Heath D,  Harris P,  Castillo Y,  Kruger H,  Arias-Stella J (1971). The carotid body in animals at high altitude. J. Pathol.

[35] Saldana MJ,  Salem LE,  Travezan R (1973). High altitude hypoxia and chemodectomas. Hum. Pathol.

[36] Nurse CA,  Vollmer C (1997). Role of basic FGF and oxygen in control of proliferation, survival, and neuronal differentiation in carotid body chromaffin cells. Dev. Biol.

[37] Lack EE,  Cubilla AL,  Woodruff JM (1979). Paragangliomas of the head and neck region. A pathologic study of tumors from 71 patients. Hum. Pathol.

[38] Lack EE,  Perez-Atayde AR,  Young JB (1985). Carotid body hyperplasia in cystic fibrosis and cyanotic heart disease. A combined morphometric, ultrastructural, and biochemical study. Am. J. Pathol.

[39] Stiller D,  Katenkamp D,  Kuttner K (1975). Jugular body tumors: hyperplasias or true neoplasms? Light and electron microscopical investigations. Virchows Arch. A Pathol. Anat. Histol.

[40] Briere JJ,  Favier J,  Benit P,  El Ghouzzi V,  Lorenzato A,  Rabier D,  Di Renzo MF,  Gimenez-Roqueplo AP,  Rustin P (2005). Mitochondrial succinate is instrumental for HIF1alpha nuclear translocation in SDHA-mutant fibroblasts under normoxic conditions. Hum. Mol. Genet.

[41] Ishii T,  Yasuda K,  Akatsuka A,  Hino O,  Hartman PS,  Ishii N (2005). A mutation in the SDHC gene of complex II increases oxidative stress, resulting in apoptosis and tumorigenesis. Cancer Res.

[42] Slane BG,  Aykin-Burns N,  Smith BJ,  Kalen AL,  Goswami PC,  Domann FE,  Spitz DR (2006). Mutation of succinate dehydrogenase subunit C results in increased O2 oxidative stress, and genomic instability. Cancer Res.

[43] Niemann S,  Muller U (2000). Mutations in SDHC cause autosomal dominant paraganglioma, type 3. Nat. Genet.

[44] Baysal BE,  Willett-Brozick JE,  Lawrence EC,  Drovdlic CM,  Savul SA,  McLeod DR,  Yee HA,  Brackmann DE,  Slattery W.H 3rd, Myers EN,  Ferrell RE,  Rubinstein WS (2002). Prevalence of SDHB, SDHC, and SDHD germline mutations in clinic patients with head and neck paragangliomas. J. Med. Genet.

[45] Astuti D,  Latif F,  Dallol A,  Dahia PL,  Douglas F,  George E,  Skoldberg F,  Husebye ES,  Eng C,  Maher ER (2001). Gene mutations in the succinate dehydrogenase subunit SDHB cause susceptibility to familial pheochromocytoma and to familial paraganglioma. Am. J. Hum. Genet.

[46] Vanharanta S,  Buchta M,  McWhinney SR,  Virta SK,  Peczkowska M,  Morrison CD,  Lehtonen R,  Januszewicz A,  Jarvinen H,  Juhola M,  Mecklin JP,  Pukkala E,  Herva R,  Kiuru M,  Nupponen NN,  Aaltonen LA,  Neumann HP,  Eng C (2004). Early-onset renal cell carcinoma as a novel extraparaganglial component of SDHB-associated heritable paraganglioma. Am. J. Hum. Genet.

[47] Ricketts C,  Woodward ER,  Killick P,  Morris MR,  Astuti D,  Latif F,  Maher ER (2008). Germline SDHB mutations and familial renal cell carcinoma. J. Natl. Cancer Inst.

[48] Ni Y,  Zbuk KM,  Sadler T,  Patocs A,  Lobo G,  Edelman E,  Platzer P,  Orloff MS,  Waite KA,  Eng C (2008). Germline mutations and variants in the succinate dehydrogenase genes in Cowden and Cowden-like syndromes. Am. J. Hum. Genet.

[49] Gellera C,  Uziel G,  Rimoldi M,  Zeviani M,  Laverda A,  Carrara F,  DiDonato S (1990). Fumarase deficiency is an autosomal recessive encephalopathy affecting both the mitochondrial and the cytosolic enzymes. Neurology.

[50] Liochev SI,  Fridovich I, Fumarase C (1992). the stable fumarase of Escherichia coli, is controlled by the soxRS regulon. Proc. Natl. Acad. Sci. USA.

[51] Bourgeron T,  Chretien D,  Poggi-Bach J,  Doonan S,  Rabier D,  Letouze P,  Munnich A,  Rotig A,  Landrieu P,  Rustin P (1994). Mutation of the fumarase gene in two siblings with progressive encephalopathy and fumarase deficiency. J. Clin. Invest.

[52] Weaver T,  Lees M,  Banaszak L (1997). Mutations of fumarase that distinguish between the active site and a nearby dicarboxylic acid binding site. Protein Sci.

[53] Tomlinson IP,  Alam NA,  Rowan AJ,  Barclay E,  Jaeger EE,  Kelsell D,  Leigh I,  Gorman P,  Lamlum H,  Rahman S,  Roylance RR,  Olpin S,  Bevan S,  Barker K,  Hearle N,  Houlston RS,  Kiuru M,  Lehtonen R,  Karhu A,  Vilkki S,  Laiho P,  Eklund C,  Vierimaa O,  Aittomaki K,  Hietala M,  Sistonen P,  Paetau A,  Salovaara R,  Herva R,  Launonen V,  Aaltonen LA (2002). Germline mutations in FH predispose to dominantly inherited uterine fibroids, skin leiomyomata and papillary renal cell cancer. Nat. Genet.

[54] Lehtonen R,  Kiuru M,  Vanharanta S,  Sjoberg J,  Aaltonen LM,  Aittomaki K,  Arola J,  Butzow R,  Eng C,  Husgafvel-Pursiainen K,  Isola J,  Jarvinen H,  Koivisto P,  Mecklin JP,  Peltomaki P,  Salovaara R,  Wasenius VM,  Karhu A,  Launonen V,  Nupponen NN,  Aaltonen LA (2004). Biallelic inactivation of fumarate hydratase (FH) occurs in nonsyndromic uterine leiomyomas but is rare in other tumors. Am. J. Pathol.

[55] Campuzano V,  Montermini L,  Molto MD,  Pianese L,  Cossee M,  Cavalcanti F,  Monros E,  Rodius F,  Duclos F,  Monticelli A,  Zara F,  Canizares J,  Koutnikova H,  Bidichandani SI,  Gellera C,  Brice A,  Trouillas P,  De Michele G,  Filla A,  De Frutos R,  Palau F,  Patel PI,  Di Donato S,  Mandel JL,  Cocozza S,  Koenig M,  Pandolfo M (1996). Friedreich's ataxia: autosomal recessive disease caused by an intronic GAA triplet repeat expansion. Science.

[56] Lill R,  Muhlenhoff U (2005). Iron-sulfur-protein biogenesis in eukaryotes. Trends Biochem. Sci.

[57] Rotig A,  de Lonlay P,  Chretien D,  Foury F,  Koenig M,  Sidi D,  Munnich A,  Rustin P (1997). Aconitase and mitochondrial iron-sulphur protein deficiency in Friedreich ataxia. Nat. Genet.

[58] Muhlenhoff U,  Richhardt N,  Ristow M,  Kispal G,  Lill R (2002). The yeast frataxin homolog Yfh1p plays a specific role in the maturation of cellular Fe/S proteins. Hum. Mol. Genet.

[59] Puccio H,  Simon D,  Cossee M,  Criqui-Filipe P,  Tiziano F,  Melki J,  Hindelang C,  Matyas R,  Rustin P,  Koenig M (2001). Mouse models for Friedreich ataxia exhibit cardiomyopathy, sensory nerve defect and Fe-S enzyme deficiency followed by intramitochondrial iron deposits. Nat. Genet.

[60] Shoichet SA,  Baumer AT,  Stamenkovic D,  Sauer H,  Pfeif-fer AF,  Kahn CR,  Muller-Wieland D,  Richter C,  Ristow M (2002). Frataxin promotes antioxidant defense in a thiol-dependent manner resulting in diminished malignant transformation *in vitro*. Hum. Mol. Genet.

[61] Schulz TJ,  Thierbach R,  Voigt A,  Drewes G,  Mietzner B,  Steinberg P,  Pfeiffer AF,  Ristow M (2006). Induction of oxidative metabolism by mitochondrial frataxin inhibits cancer growth: Otto Warburg revisited. J. Biol. Chem.

[62] Thierbach R,  Schulz TJ,  Isken F,  Voigt A,  Mietzner B,  Drewes G,  von Kleist-Retzow JC,  Wiesner RJ,  Magnuson MA,  Puccio H,  Pfeiffer AF,  Steinberg P,  Ristow M (2005). Targeted disruption of hepatic frataxin expression causes impaired mitochondrial function, decreased life span and tumor growth in mice. Hum. Mol. Genet.

[63] Awad MM,  Enslen H,  Boylan JM,  Davis RJ,  Gruppuso PA (2000). Growth regulation *via* p38 mitogen-activated protein kinase in developing liver. J. Biol. Chem.

[64] Iyoda K,  Sasaki Y,  Horimoto M,  Toyama T,  Yakushijin T,  Sakakibara M,  Takehara T,  Fujimoto J,  Hori M,  Wands JR,  Hayashi N (2003). Involvement of the p38 mitogen-activated protein kinase cascade in hepatocellular carcinoma. Cancer.

[65] Sutton A,  Imbert A,  Igoudjil A,  Descatoire V,  Cazanave S,  Pessayre D,  Degoul F (2005). The manganese superoxide dismutase Ala16Val dimorphism modulates both mitochondrial import and mRNA stability. Pharmacogenet. Genomics.

[66] Li H,  Kantoff PW,  Giovannucci E,  Leitzmann MF,  Gaziano JM,  Stampfer MJ,  Ma J (2005). Manganese superoxide dismutase polymorphism, prediagnostic antioxidant status, and risk of clinical significant prostate cancer. Cancer Res.

[67] Moschos MP (2000). Selenoprotein P. Cell. Mol. Life Sci.

[68] Meplan C,  Crosley LK,  Nicol F,  Beckett GJ,  Howie AF,  Hill KE,  Horgan G,  Mathers JC,  Arthur JR,  Hesketh JE (2007). Genetic polymorphisms in the human selenoprotein P gene determine the response of selenoprotein markers to selenium supplementation in a gender-specific manner (the SELGEN study). FASEB J.

[69] Cooper ML,  Adami HO,  Gronberg H,  Wiklund F,  Green FR,  Rayman MP (2008). Interaction between single nucleotide polymorphisms in selenoprotein P and mitochondrial superoxide dismutase determines prostate cancer risk. Cancer Res.

[70] Hirano M,  DiMauro S (2001). ANT1, Twinkle, POLG, and TP: new genes open our eyes to ophthalmoplegia. Neurology.

[71] Bolden A,  Noy GP,  Weissbach A (1977). DNA polymerase of mito-chondria is a gamma-polymerase. J. Biol. Chem.

[72] Ropp PA,  Copeland WC (1996). Cloning and characterization of the human mitochondrial DNA polymerase, DNA polymerase gamma. Genomics.

[73] Rovio AT,  Marchington DR,  Donat S,  Schuppe HC,  Abel J,  Fritsche E,  Elliott DJ,  Laippala P,  Ahola AL,  McNay D,  Harrison RF,  Hughes B,  Barrett T,  Bailey DM,  Mehmet D,  Jequier AM,  Hargreave TB,  Kao SH,  Cummins JM,  Barton DE,  Cooke HJ,  Wei YH,  Wichmann L,  Poulton J,  Jacobs HT (2001). Mutations at the mitochondrial DNA polymerase (POLG) locus associated with male infertility. Nat. Genet.

[74] Blomberg Jensen M,  Leffers H,  Petersen JH,  Daugaard G,  Skakkebaek NE,  Rajpert-De Meyts E (2008). Association of the polymorphism of the CAG repeat in the mitochondrial DNA polymerase gamma gene (POLG) with testicular germ-cell cancer. Ann. Oncol.

[75] Petros JA,  Baumann AK,  Ruiz-Pesini E,  Amin MB,  Sun CQ,  Hall J,  Lim S,  Issa MM,  Flanders WD,  Hosseini SH,  Marshall FF,  Wallace DC (2005). mtDNA mutations increase tumorigenicity in prostate cancer. Proc. Natl. Acad. Sci. USA.

[76] King MP,  Attardi G (1989). Human cells lacking mtDNA: repopulation with exogenous mitochondria by complementation. Science.

[77] Evans AR,  Limp-Foster M,  Kelley MR (2000). Going APE over ref-1. Mutat. Res.

[78] Polyak K,  Li Y,  Zhu H,  Lengauer C,  Willson JK,  Marko-witz SD,  Trush MA,  Kinzler KW,  Vogelstein B (1998). Somatic mutations of the mitochondrial genome in human colorectal tumours. Nat. Genet.

[79] Habano W,  Nakamura S,  Sugai T (1998). Microsatellite instability in the mitochondrial DNA of colorectal carcinomas: evidence for mismatch repair systems in mitochondrial genome. Oncogene.

[80] Sanchez-Cespedes M,  Parrella P,  Nomoto S,  Cohen D,  Xiao Y,  Esteller M,  Jeronimo C,  Jordan RC,  Nicol T,  Koch WM,  Schoenberg M,  Mazzarelli P,  Fazio VM,  Sidransky D (2001). Identification of a mononucleotide repeat as a major target for mitochondrial DNA alterations in human tumors. Cancer Res.

[81] Habano W,  Sugai T,  Yoshida T,  Nakamura S (1999). Mitochondrial gene mutation, but not large-scale deletion, is a feature of colorectal carcinomas with mitochondrial microsatellite instability. Int. J. Cancer.

[82] Parrella P,  Xiao Y,  Fliss M,  Sanchez-Cespedes M,  Mazzarelli P,  Rinaldi M,  Nicol T,  Gabrielson E,  Cuomo C,  Cohen D,  Pandit S,  Spencer M,  Rabitti C,  Fazio VM,  Sidransky D (2001). Detection of mitochondrial DNA mutations in primary breast cancer and fine-needle aspirates. Cancer Res.

[83] Zhou S,  Kachhap S,  Sun W,  Wu G,  Chuang A,  Poeta L,  Grumbine L,  Mithani SK,  Chatterjee A,  Koch W,  Westra WH,  Maitra A,  Glazer C,  Carducci M,  Sidransky D,  McFate T,  Verma A,  Califano JA (2007). Frequency and phenotypic implications of mitochondrial DNA mutations in human squamous cell cancers of the head and neck. Proc. Natl. Acad. Sci. USA.

[84] Fliss MS,  Usadel H,  Caballero OL,  Wu L,  Buta MR,  Eleff SM,  Jen J,  Sidransky D (2000). Facile detection of mito-chondrial DNA mutations in tumors and bodily fluids. Science.

[85] Kirches E,  Michael M,  Woy C,  Schneider T,  Warich-Kirches M,  Schneider-Stock R,  Winkler K,  Wittig H,  Dietzmann K (1999). Loss of heteroplasmy in the displacement loop of brain mitochondrial DNA in astrocytic tumors. Genes Chromosomes Cancer.

[86] Kirches E,  Krause G,  Warich-Kirches M,  Weis S,  Schneider T,  Meyer-Puttlitz B,  Mawrin C,  Dietzmann K (2001). High frequency of mitochondrial DNA mutations in glioblastoma multiforme identified by direct sequence comparison to blood sam-ples. Int. J. Cancer.

[87] Montanini L,  Regna-Gladin C,  Eoli M,  Albarosa R,  Carrara F,  Zeviani M,  Bruzzone MG,  Broggi G,  Boiardi A,  Finocchiaro G (2005). Instability of mitochondrial DNA and MRI and clinical correlations in malignant gliomas. J. Neurooncol.

[88] Kirches E,  Mawrin C,  Schneider-Stock R,  Krause G,  Scherlach C,  Dietzmann K (2003). Mitochondrial DNA as a clonal tumor cell marker: gliomatosis cerebri. J. Neurooncol.

[89] Wong LJ,  Lueth M,  Li XN,  Lau CC,  Vogel H (2003). Detection of mitochondrial DNA mutations in the tumor and cerebrospinal fluid of medulloblastoma patients. Cancer Res.

[90] Jenuth JP,  Peterson AC,  Shoubridge EA (1997). Tissue-specific selection for different mtDNA genotypes in heteroplasmic mice. Nat. Genet.

[91] Habano W,  Sugai T,  Nakamura SI,  Uesugi N,  Yoshida T,  Sasou S (2000). Microsatellite instability and mutation of mitochondrial and nuclear DNA in gastric carcinoma. Gastroenterology.

[92] Coller HA,  Khrapko K,  Bodyak ND,  Nekhaeva E,  Herrero-Jimenez P,  Thilly WG (2001). High frequency of homoplasmic mitochondrial DNA mutations in human tumors can be explained without selection. Nat. Genet.

[93] Matsuyama W,  Nakagawa M,  Wakimoto J,  Hirotsu Y,  Kawabata M,  Osame M (2003). Mitochondrial DNA mutation correlates with stage progression and prognosis in non-small cell lung cancer. Hum. Mutat.

[94] Mountain CF (1997). Revisions in the International System for Staging Lung Cancer. Chest.

[95] Lievre A,  Chapusot C,  Bouvier AM,  Zinzindohoue F,  Piard F,  Roignot P,  Arnould L,  Beaune P,  Faivre J,  Laurent-Puig P (2005). Clinical value of mitochondrial mutations in colorectal cancer. J. Clin. Oncol.

[96] Ha PK,  Tong BC,  Westra WH,  Sanchez-Cespedes M,  Parrella P,  Zahurak M,  Sidransky D,  Califano JA (2002). Mitochondrial C-tract alteration in premalignant lesions of the head and neck: a marker for progression and clonal proliferation. Clin. Cancer Res.

[97] Isaacs C,  Cavalli LR,  Cohen Y,  Pennanen M,  Shankar LK,  Freedman M,  Singh B,  Liu M,  Gallagher A,  Rone JD,  Dickson RB,  Sidransky D,  Haddad BR (2004). Detection of LOH and mitochondrial DNA alterations in ductal lavage and nipple aspirate fluids from hngh-risk patients. Breast Cancer Res. Treat.

[98] Maitra A,  Cohen Y,  Gillespie SE,  Mambo E,  Fukushima N,  Hoque MO,  Shah N,  Goggins M,  Califano J,  Sidransky D,  Chakravarti A (2004). The Human MitoChip: a high-throughput sequencing microarray for mitochondrial mutation detection. Genome Res.

[99] Zhou S,  Kassauei K,  Cutler DJ,  Kennedy GC,  Sidransky D,  Maitra A,  Califano J (2006). An oligonucleotide microarray for high-throughput sequencing of the mitochondrial genome. J. Mol. Diagn.

[100] Sui G,  Zhou S,  Wang J,  Canto M,  Lee EE,  Eshleman JR,  Montgomery EA,  Sidransky D,  Califano JA,  Maitra A (2006). Mitochondrial DNA mutations in preneoplastic lesions of the gastrointestinal tract: a biomarker for the early detection of cancer. Mol. Cancer.

[101] Jakupciak JP,  Maragh S,  Markowitz ME,  Greenberg AK,  Hoque MO,  Maitra A,  Barker PE,  Wagner PD,  Rom WN,  Srivastava S,  Sidransky D,  O'Connell CD (2008). Performance of mitochondrial DNA mutations detecting early stage cancer. BMC Cancer.

